# Frequent Travelers and Rate of Spread of Epidemics

**DOI:** 10.3201/eid1309.070081

**Published:** 2007-09

**Authors:** T. Déirdre Hollingsworth, Neil M. Ferguson, Roy M. Anderson

**Affiliations:** * Imperial College London, London, United Kingdom

**Keywords:** SARS, influenza, epidemics, mathematical model, international air travel, research

## Abstract

A small proportion of air travelers make disproportionately more journeys than the rest of travelers. They also tend to interact predominantly with other frequent travelers in hotels and airport lounges. This group has the potential to accelerate global spread of infectious respiratory diseases. Using an epidemiologic model, we simulated exportation of cases from severe acute respiratory syndrome–like and influenza-like epidemics in a population for which a small proportion travel more frequently than the rest. Our simulations show that frequent travelers accelerate international spread of epidemics only if they are infected early in an outbreak and the outbreak does not expand rapidly. If the epidemic growth rate is high, as is likely for pandemic influenza, heterogeneities in travel are frequently overwhelmed by the large number of infected persons in the majority population and the resulting high probability that some of these persons will take an international flight.

## Abstract 2

These travelers may increase spread of epidemics that have a long generation time, but have little effect on fast-spreading epidemics.

In today’s world of increasing air travel for both business and pleasure, a small proportion of persons make disproportionately more journeys than the rest of the population (*1*,*2*). These frequent fliers tend to travel for business purposes and mix predominantly with other business travelers, stay in particular hotels, and use specific airport lounges. This form of assortative (like with like) mixing means a respiratory infection could potentially spread quickly within this group and thus be disseminated rapidly between countries. This rapid spread was illustrated early in the severe acute respiratory syndrome (SARS) outbreak of 2003. The index SARS case in Hong Kong Special Administrative Region, People’s Republic of China, stayed in a hotel and infected 16 persons there. Of these patients with secondary cases, 6 took international flights to Australia, Canada, Singapore, the Philippines, and Vietnam (*3*). The arrival of these infected persons subsequently led to SARS outbreaks in Hanoi, Singapore, and Toronto within a few days of the first case in Hong Kong.

Recent studies of the role of international air travel on the spread of infectious diseases have highlighted the role of heterogeneities in the connectedness of different airports (*4*–*6*), the length of the latent period of the disease in relation to the duration of the flight (*7*), the possible role of travel restrictions (*8*–*11*) and the role of cooperative strategies to control international spread of pandemic influenza (*10*,*12*). To date, none of these studies has taken into account the effects of heterogeneity in the frequency of travel between persons and the potential role of such heterogeneity on the global spread of a directly transmitted infectious agent. Also of interest is whether targeting interventions specifically at frequent travelers would slow the international spread of a defined pathogen.

## Methods

To investigate the role of frequent travelers in the exportation of asymptomatic cases during the early stages of an epidemic, we simulated outbreaks of both a SARS-like and an influenza-like airborne respiratory infection in a population in which a small proportion of the population make many more trips than the rest of the population. In the early stages of an epidemic, chance events are important because the number of infected persons is small. We simulated these early stages by using a stochastic model for which every simulation is different. We present both the mean behavior of the simulations and the range of possible outcomes across a large number of simulations. In a stochastic model, introduction of 1 infected person has a finite probability of resulting in the rapid extinction of an infectious disease. To increase the probability of initiating an outbreak, we introduced 3 asymptomatic persons into the population. We simulated the outbreak in a large extended metropolitan area with a population of 10^7^ persons.

**Figure 1:**
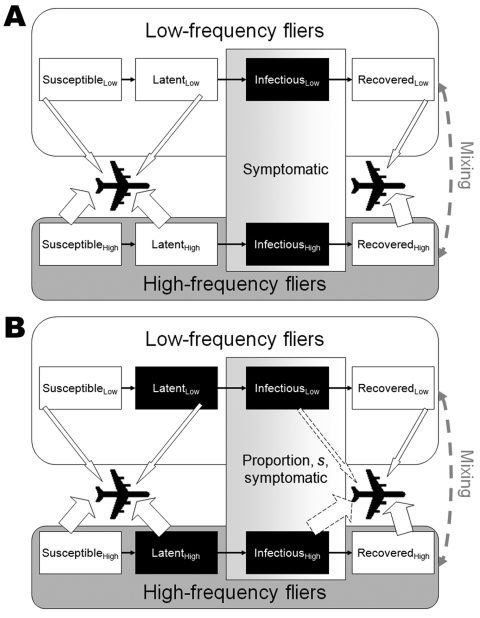
Schematic representation of the model structure. Black boxes represent infectious stages and arrows indicate that persons in these populations are allowed to fly. A) Severe acute respiratory syndrome. Persons with latent infections are not infectious, and all infectious persons are symptomatic and prevented from traveling. B) Pandemic influenza. Persons with latent infections are infectious, and a proportion (1 – *s*) of infectious persons are asymptomatic and allowed to travel (indicated by the dotted arrows). The size of the arrows indicates that the persons in the high-frequency flier group have a higher probability of flying per day.

The structure of the model is illustrated schematically in Figure 1A. The population is divided into 2 subpopulations with different frequencies of taking international flights. A small proportion of the population, *r*, are high-frequency fliers. Most of the population, 1 – *r*, are low-frequency fliers. Frequent fliers have contact with other frequent fliers and with the general population. Similarly, the general population has contact with persons in the general population and with frequent fliers. Contacts are more likely to be between persons within each group (frequent fliers or general population), but the level of this assortativeness may vary (parameterized by Φ). Contacts may be made completely randomly, with the likelihood of meeting a person from the frequent-flying group or the general population being proportional to the number of persons in each population (Φ = 1). At the other extreme, persons may only have contact with other persons in the same group (Φ = 0). The true mixing pattern is likely to lie between these 2 extremes.

The extent to which the high-frequency and low-frequency fliers mix will determine how quickly a disease will spread from the general population to the frequent fliers and vice versa. We simulated the model for a selection of mixing parameters, ranging from wholly random (Φ = 1) to moderate and high levels of assortativeness (Φ = 0.5, 0.25, respectively). For comparison, we also simulated a homogeneous model in which the entire population travels equally frequently.

The outbreak is modeled by dividing the population into those who are still susceptible to the disease, those who have contracted the disease and are in the latent stage, those who are infectious and symptomatic, and those who have recovered from the disease (Figure 1). This division is similar to the basic structure used in several recent papers on the role of international travel in the spread of infectious diseases (*8*,*9*,*12*). This model structure can be adapted to many airborne infections because it allows for an asymptomatic period, which may or may not be infectious, followed by a potentially symptomatic period during which transmission can also occur.

In our stochastic model, events (such as infection or a person leaving the source area) occur by chance. For example, the time after symptom onset at which a person recovers from infection with SARS is not a fixed quantity; rather, it is a randomly chosen time with a mean of 10 days. Table 1 shows the average latent and infectious periods used. The probability of leaving the country is constant for all persons (Table 1). The probability of a susceptible person becoming infected increases as a larger proportion of the population becomes infected and is chosen so that the average number of new infections caused by each infected person in the early stages of the epidemic is equal to the basic reproductive number *R_0_* (2.5 for SARS, 1.8 for influenza; Table 1). The epidemic is simulated by evaluating the probability that any person is infected, becomes symptomatic, or recovers in any short time interval (we divide time into sequential short intervals of one fiftieth of a day), and then testing whether that event occurs. The simulation can be thought of as generating a random number between 0 and 1 for each person in each time step. If this random number is less than the probability of a particular event occurring to that person, then the event occurs. Otherwise, the person is left in his or her current state. The model does not store the details of every person separately but keeps track of the number of persons who are susceptible (*S*), latently infected (*E*), infectious (*I*), and recovered (*R*) at any point in time. As events occur, these variables change. For example, when a person becomes infected, *S* decreases by 1 and *E* increases by 1. Because the events occur by chance, the total number of persons who are in each state, including the number of infected persons taking flights, varies stochastically.

**Table 1:** Parameter descriptions and values of epidemiologic model that simulates exportation of cases from SARS-like and influenza-like epidemics*

Description	Parameter	Value (reference)	
		SARS	Influenza
Infection			
Basic reproductive number	*R_0_*	2.5 (*13*)	1.8 (*14*)
Latent period, d	*T_L_*	4 (*13*)	1.5 (*14*)
Infectious period, d	*T_I_*	10 (*13*)	1.1 (*14*)
Generation time, d	*T_g_* = *T_L_* + *T_I_*	14	2.6
Epidemic doubling time, d	*t_d_* = *T_g_* / (*R_0_* – 1) ln2	6.5	2.3
International travel			
Proportion of population who are high-frequency fliers	*r*	0–0.5	
Mixing between groups: Φ = 1, random mixing; Φ = 0, assortative mixing	Φ	0–1	
Relative probability of flying of high-frequency fliers	*f*	20	
Mean probability of flying per day	*ε*	0.005 (*9*)	
Probability of flying per day of high-frequency fliers	ε*_H_* = *f* / 1 + (*f* – 1)*r* ε	0.084	
Probability of flying per day of low-frequency fliers	ε*_L_* = 1 / 1 + (*f* – 1)*r* ε	0.042	
Probability of a case being exported			
Homogeneous flying patterns	*L* = *T_L_*ε	0.02	0.008
High-frequency fliers	*l_H_* = *T_L_*ε*_H_*	0.34	0.13
Low-frequency fliers	*l_L_* = *T_L_*ε*_L_*	0.017	0.006

*SARS, severe acute respiratory syndrome.

In our model, we assume that those who are in the latent stage of the disease are not infectious for SARS and influenza. This is generally accepted to be a good model for SARS because isolation of symptomatic persons prevented onward transmission of SARS, which indicated that the latent period has limited or no infectivity (*15*). We also assume that all infectious persons are symptomatic. This is a conservative assumption, but serosurveillance studies for SARS have shown low prevalence of seropositivity in persons who did not show symptoms of disease (*16*–*21*). Lastly, we assume that all symptomatic persons are prevented from traveling because of symptom severity or effective screening. The model equations are shown in the Technical Appendix.

The disease course of a possible future influenza pandemic is not known. However, studies of previous pandemics and seasonal epidemics suggest a possible scenario in which the latent period of influenza may be infectious and not all infected persons will show symptoms (*14*,*22*–*24*). This means that a larger proportion of cases could be allowed to travel on international flights, even with 100% effective screening, because they are asymptomatically infected (Figure 1, panel B). We have modeled a conservative scenario, in which influenza has a disease life history similar to that of SARS, but with shorter latent and infectious periods (Table 1). The inclusion of partially effective screening or, equivalently, the inclusion of asymptomatic cases would lead to more cases being exported than is shown here.

Little data are available across a population for the relative frequency of flying. The mean probability of flying for the whole population can be approximated by the number of airline passengers divided by the population of a country or city. This calculation gives estimates of 0.005 for Hong Kong, 0.0005 for Beijing, and 0.0002 for Thailand (*9*). We modeled a population of 10 million persons with a 0.005 probability of flying per day as an example of an outbreak in a well-connected city. A study on domestic flying in Norway suggested that ≈2% of a survey population who take domestic flights in Norway make >20 journeys a year (*1*). This survey did not include persons who do not take flights. Therefore, the proportion of the total population who make this many journeys is likely to be lower. On the basis of this data, we present results for a population in which 1% of the population travel 20× more frequently than the rest of the population and discuss results for different values of these parameters.

We investigated the effect of the setting where the outbreak is initiated by using 2 scenarios. In the first scenario, the outbreak begins among the general, infrequently flying population. Cases subsequently occur among high-frequency fliers as a result of contact between the 2 subpopulations. The mean time until the first high-frequency flier becomes infected is a function of incidence rate in the main population and level of mixing between the 2 groups. In the second scenario, the outbreak begins among the high-frequency fliers. The disease again spreads to the main population because of contacts between the groups, with the mean time until this occurs being a function of the incidence rate in the main population and the level of mixing between the 2 groups.

The mean cumulative number of cases exported (across 50,000 simulations) is presented for both SARS-like and influenza-like parameters (Table 1), for initiation of the epidemic among the low-frequency and high-frequency fliers, and for a range of mixing between the high-frequency and low- frequency travelers. We also illustrate variability in simulated outcomes by presenting snapshots of the distributions of the cumulative number of exported cases.

## Results

**Figure 2:**
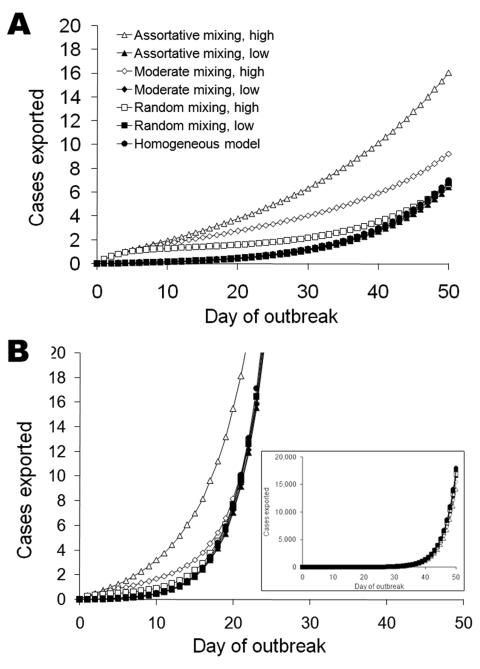
Mean number of cases exported from a single simulated source epidemic for severe acute respiratory syndrome–like parameters (A) and influenza-like parameters (B) (50,000 runs; parameters are listed in Table 1). Results are shown for a population in which everyone travels equally frequently (homogeneous model, circles), for a population in which 1% travel 20 times more frequently than the rest of the population, and for the 2 populations mixed randomly (Φ = 1, squares) for moderate levels of mixing between the groups (Φ = 0.5, diamonds) and for low levels of mixing in which most contacts are assortative (Φ = 0.25, triangles). The first cases are either in the majority population of low-frequency fliers (solid symbols) or high-frequency fliers (open symbols). Inset in B shows a greater range on the y-axis. Variability about these means is shown in Table 2 and Appendix Figure 1.

As an epidemic progresses, the cumulative number of cases increases, and therefore the number of asymptomatic cases exported from a source area increases for all travel patterns (Figure 2). If a SARS-like epidemic is seeded in the group of frequent fliers, then the initial rate of international spread is accelerated relative to the rate for the homogeneous case (Figure 2, panel A, open symbols). If the frequent travelers contract the infection early, more exclusivity of mixing (smaller Φ) serves to speed international spread, and this effect may last well into the epidemic (Figure 2, panel A, open triangles). If the epidemic is initiated in the low-frequency fliers, the mean number of exported cases is similar to results of the homogeneous model (Figure 2, panel A, closed symbols). Heterogeneities in travel patterns increase the variability between simulated epidemics; higher variability results from more assortative mixing (Table 2; Appendix Figure 1.

**Table 2:** Variability between runs in an epidemiologic model that simulates exportation of cases from SARS-like and influenza-like epidemics*

Mixing pattern	First case	No. cases exported, mean, median (5th–95th percentile)				
		Day 10	Day 20	Day 30	Day 40	Day 50
SARS						
Homogeneous flying patterns		0, 0 (0–0)	0, 0 (0–1)	1, 0 (0–3)	3, 1 (0–7)	7, 5 (1–16)
Random mixing	High	1, 0 (0–2)	2, 0 (0–2)	2, 1 (0–3)	4, 2 (1–7)	7, 5 (2–14)
	Low	0, 0 (0–0)	0, 0 (0–1)	1, 0 (0–3)	3, 1 (0–6)	7, 5 (1–15)
Moderately assortative	High	2, 1 (0–3)	3, 2 (1–4)	4 (3, 1–7)	6 (4, 2–12)	9, 7 (2–20)
	Low	0, 0 (0–0)	0, 0 (0–1)	1, 0 (0–2)	3, 1 (0–6)	7, 5 (0–15)
Highly assortative	High	2, 1 (0–3)	4, 2 (1–7)	5, 5 (2–13)	10, 8 (3–22)	16, 12 (4–38)
	Low	0, 0 (0–0)	0, 0 (0–1)	1, 0 (0–2)	3, 1 (0–6)	6, 4 (1–15)
Influenza						
Homogeneous flying patterns		1, 0 (0–1)	8, 5 (0–20)	107, 85 (1–251)	1,268, 1,069 (7–3,118)	15,729, 13,541 (73–35,132)
Random mixing	High	1, 0 (0–2)	7, 5 (0–18)	89, 74 (1–233)	1,341, 940 (1–3,049)	14,592, 11,990 (1–35,632)
	Low	0, 0 (0–1)	7, 5 (0–18)	95, 78 (1– 246)	1,264, 1,057 (7–3,256)	15,668, 13,651 (74– 35,231)
Moderately assortative	High	2, 0 (0–3)	8, 6 (0–32)	93, 72 (1–231)	1,288, 1,138 (1–3,387)	15,505, 14,362 (1–32,134)
	Low	1, 0 (0–1)	7, 5 (0–20)	104, 83 (0–264)	1,411, 1,213 (0–3,526)	17,081, 15,850 (0–35,403)
Highly assortative	High	3, 2 (0–7)	15, 10 (2–41)	106, 81 (2–291)	1,166, 840 (2–2,923)	14,145, 10,770 (2–34,351)
	Low	0, 0 (0–2)	12, 0 (0–33)	164, 139 (0–246)	1,312, 967 (1–3,231)	16,592, 12,607 (28–36,643)
*Means are shown in Figure 2. SARS, severe acute respiratory syndrome.						

In an outbreak in which the infection spreads rapidly, such as could potentially occur with pandemic influenza A (Figure 2, panel B), heterogeneities in travel patterns have less effect on the rate of exportation of cases early in the epidemic than they would for SARS (Figure 2, panel A), particularly after the first weeks of the epidemic. The overall pattern of the exportation of cases is similar for SARS and influenza, but the time scale for influenza is much shorter because of the short doubling time (Table 1). For example, the number of exported cases is in the thousands for influenza by day 50 (Figure 2, panel B), when it is <20 for SARS (Figure 2, panel A).

Later in an epidemic, the mean number of exported cases is similar, regardless of where the epidemic is seeded or the mixing patterns of the high-frequency fliers and low-frequency fliers (Figure 2, panel B, inset for influenza, not shown for SARS). The variability between simulated epidemics becomes large, with some simulations resulting in hundreds of exported cases and many resulting in only a few exported cases (Table 2; Appendix Figure 1, panel D).

Heterogeneities in travel patterns increase the number of exported cases to a greater extent and for a longer period if the relative frequency of flying of the high-frequency fliers, *f*, is higher or if the proportion of the population who are high-frequency fliers, *r*, is smaller (Appendix Figure 2) because the probability that any frequent flier will fly per day is higher (Table 2, ε*_H_*). However, if *r* becomes small, the epidemic among this group peaks and then decreases quickly because of the limited number in the group. In this case, the period in which there are enough infected persons in this group who can contribute to an increased rate of spread of exportation of cases is short (Appendix Figure 2, panel B).

## Discussion

The probability that an infected person will make an international flight while still incubating infection and nonsymptomatic is higher for a high-frequency flier than for a low-frequency flier (Table 1). In the early stages of an epidemic in which most cases occur in high-frequency fliers, the expected number of cases exported will therefore be higher than if the early cases occur in predominantly low-frequency fliers (Figure 2). Heterogeneity in flying patterns also increases the variability between simulated outbreaks (Table 2; Appendix Figure 1).

Wherever the epidemic is initially concentrated, the disease will spread to all parts of the population because of contacts between persons in both groups. The speed with which this occurs will be a function of the level of mixing between the groups. If high-frequency fliers mix almost exclusively among themselves, they are unlikely to acquire cases early in an epidemic in which the first cases emerge in the general population. If, however, they contract the infection early, this exclusivity serves to speed international spread and this effect may last well into the epidemic (Figure 2, open triangles). If mixing is less assortative, then the epidemic will spread to the general population more rapidly. Because most of the population are low-frequency fliers, the number of infected persons in the main population will quickly exceed those in the small group of high-frequency fliers.

When the number of cases becomes large, the expected number of exported cases indicates that the expected number of exported cases (which may be approximated as the probability of flying while asymptomatic multiplied by cumulative incidence [*9*]) will be large, even if the probability that any person travels is small. Once the epidemic takes hold in the general population, the number of cases being exported from the majority low-frequency flier population exceeds those being exported from the much smaller group of high-frequency fliers. Regardless of where most initial cases occur, the contribution of high-frequency fliers to international spread is eventually overwhelmed by the large epidemic in the general population, despite their lower probability of flying per day. Thus, the average behavior of epidemics is eventually similar, whether they start in high-frequency fliers, or in groups with no heterogeneities in travel (Figure 2, panel B, inset), but the variability between simulations is large (Table 2; Appendix Figure 1).

The latent period for influenza is likely to be shorter than that for SARS, which reduces the probability that any infected person will travel before exhibiting symptoms (Table 1). However, the doubling time for an influenza pandemic is less than half that for SARS because of the much shorter generation time for influenza (Table 1). Therefore, the number of cases exported from a local influenza epidemic will increase far more rapidly than those from a SARS epidemic (Figure 2, panel B). This rapid growth means that any increased rate of export caused by early concentration of infection among the high-frequency fliers will be quickly overcome by the number of cases being exported from the general population (Figure 2, panel B), which indicates that heterogeneities in travel have little effect.

We have simulated an outbreak in a single population by using a relatively simple model. Similar models have been used for the dynamics of single epidemics in a network of countries or areas connected by a complex airline network (*6*,*8*,*12*), and more complex, person-based, within-country models have been used to simulate epidemics within smaller groups of countries (*10*,*14*). Our results show that in the event of an influenza pandemic, interventions such as travel restrictions will have to be implemented rapidly and effectively to have a substantial effect (*8*–*10*,*12*). We have shown that high-frequency fliers have the potential to spread infection even more rapidly than previously indicated by models that assume homogenous travel behavior.

Our study and the relatively simple structure of the model were limited by the lack of available data on the travel patterns of persons. Travel patterns may vary with age, sex, occupation, and district or country of origin. To increase our knowledge of these patterns, existing surveys of airline passengers at airports could be extended to ask additional questions on number of journeys per year. However, these surveys would necessarily omit those persons who do not take international flights, who are believed to make up a large proportion of many populations. Any additional information could be valuable for assessing the risk for international spread of diseases from affected areas.

The SARS epidemic in Hong Kong satisfied the criteria we have identified for frequent travelers, which accelerated international spread of an outbreak. The first case-patient with SARS in Hong Kong had contact with other frequent travelers in a hotel and seeded the epidemic in high-frequency travelers. However, SARS has long incubation and infectious periods and only moderate transmissibility. For influenza A, which has much shorter incubation and infectious periods, such heterogeneities have a limited effect on the rate of exportation of cases. Because frequent travelers play a role mainly in the early stages of an epidemic, targeting interventions to these persons is unlikely to be an effective control strategy because such a plan would have to be in place almost immediately.

Finally, estimates of the rate of international spread of respiratory infections that do not consider heterogeneities in behavior may be misleading. If an outbreak begins in a rural area, where persons have a low probability of traveling abroad and mixing with frequent fliers, the time until cases are exported is longer than in outbreaks in which frequent travelers contract infection early in the course of the outbreak. When combined with the vagaries of chance early in the evolution of a new epidemic and the complexities of the international airline network, this variability makes early prediction of the pattern and speed of global spread difficult. This difficulty in predicting whether a particular country is likely to import cases from a currently unknown source area highlights the need for developing a strategy for controlling an outbreak caused by imported cases.

## Supplementary Material

Technical Appendix Model Description

Appendix Figure 1 Truncated distribution (50,000 runs) of number of cases exported from a single simulated source epidemic for severe acute respiratory syndrome–like parameters (A and B) and influenza-like parameters (C and D) (50,000 runs, parameters are listed in Table 1) on day 10 (A and C) and day 20 (B and D) after introduction of the first cases. Results are shown for a population in which everyone travels equally frequently, (homogeneous model, circles), for a population in which 1% travels 20 times more frequently than the rest of the population, and for the 2 populations mixed randomly (Φ = 1, squares) for moderate levels of mixing between the groups (Φ = 0.5, diamonds) and for low levels of mixing, in which most contacts are assortative (Φ = 0.25, triangles). The first cases are either in the majority population of low-frequency fliers (solid symbols) or the high-frequency fliers (open symbols).

Appendix Figure 2 Mean number of exported cases from 50,000 simulations. Parameters are as in Figure 2, with assortative mixing (Φ = 0.25) and initiating the epidemic among high-frequency fliers. A ) Effect of varying relative frequency of flying in high-frequency travelers with that in low-frequency fliers (*f =* 1, 10, 20, and 100. B) Effect of varying proportion of the population in the high-frequency traveling group (*r =* 0.000001, 0.00001, 0.0001, 0.001, 0.01, and 0.1.
