# Supplementary material for: Frequent Travelers and Rate of Spread of Epidemics
Source: Emerg Infect Dis. 2007 Sep;13(9):1288–94. doi: 10.3201/eid1309.070081 (PMC2857283; doi:10.3201/eid1309.070081)
Supplement: Technical Appendix — Model Description [file 07-0081_Techapp-s1.pdf]

Publisher: CDC; Journal: Emerging Infectious Diseases
Article Type: Research; Volume: 13; Issue: 9; Year: 2007; Article ID: 07-0081.techapp
DOI: 10.3201/eid1309.070081; TOC Head: Research

Technical Appendix

Model Description

We use a synchronous stochastic SEIR model (susceptible, *S*; latent, *E*; infectious, *I*; recovered, *R*) model. We assume cases can only be exported from among the latently infected persons and not from symptomatic persons due to 100% effective screening. We do not model the exportation of susceptible and recovered persons because we assume that the number leaving and returning on flights balances out during the early stages of an outbreak, as was seen at Hong Kong Special Administrative Region, People's Republic of China, International Airport during the severe acute respiratory syndrome epidemic (*1*).

There is a constant hazard of each particular event occurring for each person. This is equivalent to the population having exponentially distributed times from becoming infectious to recovering from disease. For parsimony, we are modeling the number of persons in each state, as opposed to tracking every person separately. Rather than generating several random numbers to test whether a particular event occurs for each person, we generate 4 random numbers in each time step that simulate the number of persons to whom each event occurs. The binomial distribution describes the number of events that occur if there are several independent trials (e.g., $I(t)$ infectious persons who could recover), each with the same probability of occurring (every person has the same probability of recovering in each time step). Numbers of each event that occur are generated randomly at each time step δt, by using a lagged Fibonaci generator in the software package Berkeley Madonna (www.berkeleymadonna.com). The homogeneous model is governed by the following equations.

Variables

$$\begin{aligned}
&\text{Susceptibles} && \mathrm{S}(t+\delta t) = S(t) - \iota(t) \\
&\text{Exposed} && \mathrm{E}(t+\delta t) = E(t) + \iota(t) - \kappa(t) - \xi(t) \\
&\text{Infectious} && \mathrm{I}(t+\delta t) = I(t) + \kappa(t) - \rho(t) \\
&\text{Recovered} && \mathrm{R}(t+\delta t) = \rho(t) \\
&\text{Total population} && \mathrm{N}(t) = S(t) + E(t) + I(t) + R(t)
\end{aligned} \tag{1}$$

Publisher: CDC; Journal: Emerging Infectious Diseases
Article Type: Research; Volume: 13; Issue: 9; Year: 2007; Article ID: 07-0081.techapp
DOI: 10.3201/eid1309.070081; TOC Head: Research

Events

$$\begin{aligned}
&\text{incidence} && \iota(\mathrm{t}) = \text{Binomial}\left(\frac{R_0}{T_I}\frac{I(t)}{N(t)}\delta t, S(t)\right) \\
&\text{infectious} && \kappa(t) = \text{Binomial}\left(\frac{1}{T_L}\delta t, E(t)\right) \\
&\text{export} && \xi(t) = \text{Binomial}\left(\frac{\varepsilon \delta t}{1-1/T_L \delta t}, E(t)-\kappa(t)\right) \\
&\text{recovery} && \rho(t) = \text{Binomial}\left(\frac{1}{T_I}\delta t, I(t)\right)
\end{aligned} \tag{2}$$

where parameters are given in Table 1 and hazards are given in the Appendix Table.

For the competing hazards of exposed persons progressing to be infectious or leaving the country, the events could either be computed by using a multinomial distribution or equivalently by the 2 binomial distributions above: the multinomial probability of $\kappa(t)$ of $E(t)$ exposed persons progressing and $\xi(t)$ leaving the country per unit time, with the hazards of each event equal for each person (Table 2), is, by definition

$$\begin{aligned}
&M\left(\kappa,\xi;1/T_L\delta t,\varepsilon\delta t,E\right) \\
&= \frac{E!}{\kappa!\xi!(E-\kappa-\xi)!}\left(\frac{1}{T_L}\delta t\right)^{\kappa}\left(\varepsilon\delta t\right)^{\xi}\left(1-\left(\frac{1}{T_L}+\varepsilon\right)\delta t\right)^{E-\kappa-\xi} \\
&= \frac{E!}{\kappa!(E-\kappa)!}\left(\frac{1}{T_L}\delta t\right)^{\kappa}\left(1-\frac{1}{T_L}\delta t\right)^{E-\kappa}\frac{(E-\kappa)!}{\xi!(E-\kappa-\xi)!}\frac{\left(\varepsilon\delta t\right)^{\xi}\left(1-\left(1/T_L+\varepsilon\right)\delta t\right)^{E-\kappa-\xi}}{\left(1-1/T_L\delta t\right)^{E-\kappa}} \\
&= B\left(\kappa;1/T_L\delta t,E\right)\frac{(E-\kappa)!}{\xi!(E-\kappa-\xi)!}\frac{\left(\varepsilon\delta t\right)^{\xi}}{\left(1-1/T_L\delta t\right)^{\xi}}\left(1-\frac{\varepsilon\delta t}{\left(1-1/T_L\delta t\right)}\right)^{E-\kappa-\xi} \\
&= B\left(\kappa;1/T_L\delta t,E\right)B\left(\xi;\frac{\varepsilon\delta t}{1-1/T_L\delta t},E-\kappa\right)
\end{aligned} \tag{3}$$

where $B(k;p,n)$ is the binomial probability of k events from n attempts, each with probability p. Thus, the number of progression and exportation events in a small time step can be simulated from the 2 binomial distributions in equation (2).

To model heterogeneous travel patterns, we divide the population into a proportion, r, who are high frequency fliers (subscript H), and a proportion, $1 - r$, who are low-frequency fliers

(subscript L). Mixing between groups ranges from wholly assortative ($\phi = 0$) to wholly random ($\phi = 1$). The subscript i takes the values H or L and the equations are as follows:

Variables

$$\begin{aligned}
&\text{Susceptibles} && S_i(t+\delta t) = S_i(t) - \iota_i(t) \\
&\text{Exposed} && E_i(t+\delta t) = E_i(t) + \iota_i(t) - \kappa_i(t) - \xi_i(t) \\
&\text{Infectious} && I_i(t+\delta t) = I_i(t) + \kappa_i(t) - \rho_i(t) \\
&\text{Recovered} && R_i(t+\delta t) = \rho_i(t) \\
&\text{Population} && N_i(t) = S_i(t) + E_i(t) + I_i(t) + R_i(t) \\
&\text{Total population} && N(t) = N_H(t) + N_L(t)
\end{aligned} \tag{4}$$

Events

$$\begin{aligned}
&\text{incidence} && \iota_H(t) = \text{Binomial}\left(\frac{R_0}{T_I}\frac{1}{N(t)}\left(\phi I_L(t) + \frac{(1-(1-r)\phi)}{r} I_H(t)\right)\delta t, S_H(t)\right) \\
& && \iota_L(t) = \text{Binomial}\left(\frac{R_0}{T_I}\frac{1}{N(t)}\left(\phi I_H(t) + \frac{(1-r\phi)}{1-r} I_L(t)\right)\delta t, S_L(t)\right) \\
&\text{infectious} && \kappa_i(t) = \text{Binomial}\left(1/T_L \delta t, E_i(t)\right) \\
&\text{export} && \xi_i(t) = \text{Binomial}\left(\frac{\varepsilon_i \delta t}{1-1/T_L \delta t}, E_i(t) - \kappa_i(t)\right) \\
&\text{recovery} && \rho_i(t) = \text{Binomial}\left(1/T_I \delta t, I_i(t)\right)
\end{aligned} \tag{5}$$

The force of infection is such that when $\phi = 0$ (wholly assortative mixing) the probability of contacting an infected person is equal to the proportion of each group who are infected

$$\iota_i(t) = \text{Binomial}\left(\frac{R_0}{T_I}\frac{I_i(t)}{N_i(t)}\delta t, S_i(t)\right) \tag{6}$$

and when $\phi = 1$ (random mixing), the probability of contacting an infected person is equal to the proportion of the *total* population who are infected.

$$\iota_i(t) = \text{Binomial}\left(\frac{R_0}{T_I}\frac{(I_H(t) + I_L(t))}{N(t)}\delta t, S_i(t)\right) \tag{7}$$

For intermediate values of ϕ, the terms are such that if the force of infection is expressed in terms of the proportion of contacts made by each person with persons in each group, ϕ_{ij}

$$\iota_H(\mathrm{t}) = \mathrm{Binomial}\left(\frac{R_0}{T_I}\left(\phi_{HH}\frac{I_H(t)}{N_H(t)} + \phi_{HL}\frac{I_L(t)}{N_L(t)}\right)\delta t, S_H(t)\right)$$
$$\iota_L(\mathrm{t}) = \mathrm{Binomial}\left(\frac{R_0}{T_I}\left(\phi_{LH}\frac{I_H(t)}{N_H(t)} + \phi_{LL}\frac{I_L(t)}{N_L(t)}\right)\delta t, S_L(t)\right) \qquad (8)$$

then the total number of contacts for persons in each group adds up to the total population as

$$\phi_{HH}N_H(t) + \phi_{HL}N_L(t) = N(t)$$
$$\phi_{LH}N_H(t) + \phi_{LL}N_L(t) = N(t) \qquad (9)$$

and contacts made between groups are symmetric, $\phi_{HL} = \phi_{LH}$. Setting $\phi_{HL} = \phi_{LH} = \phi$, we get from equation (9) that

$$\phi_{HH} = \frac{1-(1-r)\phi}{r}$$
$$\phi_{LL} = \frac{1-r\phi}{1-r} \qquad (10)$$

where we have used the relationships $r = N_H/N$ and $1 - r = N_L/N$. Using these expressions for ϕ_{HH} and ϕ_{LL} in equation (8) yields the expressions used in our SEIR model above.

Reference

<jrn>1. Hollingsworth TD, Ferguson NM, Anderson RM. Will travel restrictions control the international spread of pandemic influenza? Nat Med. 2006;12:497–9. Medline</jrn>

Technical Appendix Table. Stochastic events, hazard of each event occurring, and no. persons to whom each event could occur in the homogeneous model

Events	Flow of persons between states	Hazard of each event occurring	No. persons to whom event could occur
Incidence of infection	S→E	$R_0/T\ I/N$	S
Progression of disease	E→I	$1/T_L$	E
Recovery	I→R	$1/T_I$	I
Exportation of asymptomatic cases	E→export	ε	E
