# Supplementary figures and images for: Frequent Travelers and Rate of Spread of Epidemics
Source: Emerg Infect Dis. 2007 Sep;13(9):1288–94. doi: 10.3201/eid1309.070081 (PMC2857283; doi:10.3201/eid1309.070081)

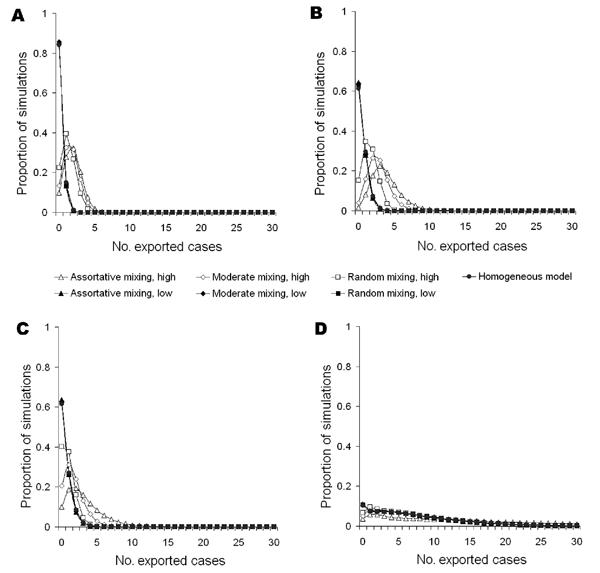

Supplement: Appendix Figure 1 — Truncated distribution (50,000 runs) of number of cases exported from a single simulated source epidemic for severe acute respiratory syndrome–like parameters (A and B) and influenza-like parameters (C and D) (50,000 runs, parameters are listed in Table 1) on day 10 (A and C) and day 20 (B and D) after introduction of the first cases. Results are shown for a population in which everyone travels equally frequently, (homogeneous model, circles), for a population in which 1% travels 20 times more frequently than the rest of the population, and for the 2 populations mixed randomly (Φ = 1, squares) for moderate levels of mixing between the groups (Φ = 0.5, diamonds) and for low levels of mixing, in which most contacts are assortative (Φ = 0.25, triangles). The first cases are either in the majority population of low-frequency fliers (solid symbols) or the high-frequency fliers (open symbols). [file 07-0081_appF1-s2.gif]

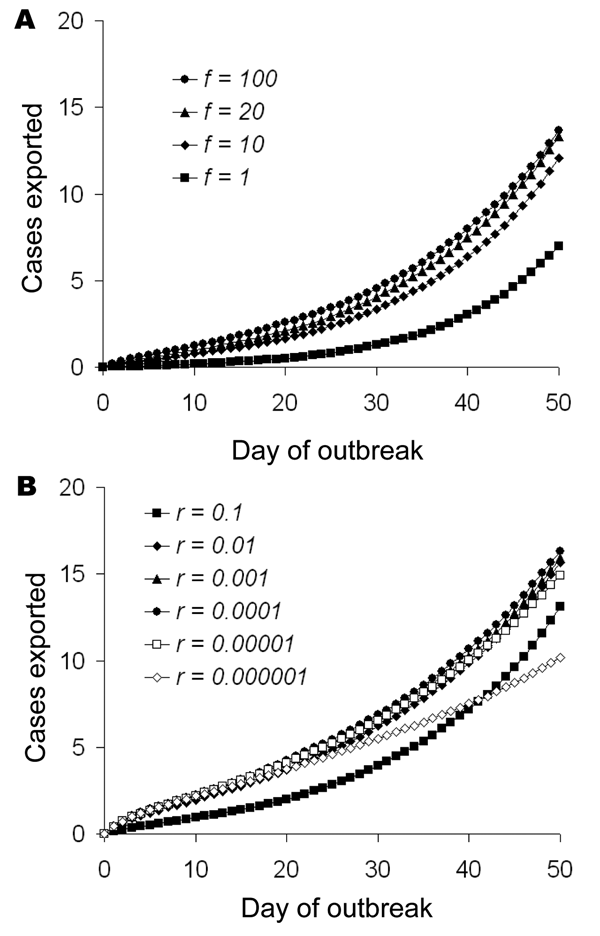

Supplement: Appendix Figure 2 — Mean number of exported cases from 50,000 simulations. Parameters are as in Figure 2, with assortative mixing (Φ = 0.25) and initiating the epidemic among high-frequency fliers. A ) Effect of varying relative frequency of flying in high-frequency travelers with that in low-frequency fliers (f = 1, 10, 20, and 100. B) Effect of varying proportion of the population in the high-frequency traveling group (r = 0.000001, 0.00001, 0.0001, 0.001, 0.01, and 0.1. [file 07-0081_appF2-s3.gif]
